# Socio-demographic factors and availability of piped fountains affect food hygiene practice of food handlers in Bahir Dar Town, northwest Ethiopia: a cross-sectional study

**DOI:** 10.1186/s13104-017-2965-2

**Published:** 2017-11-28

**Authors:** Terefe Derso, Amare Tariku, Fekadu Ambaw, Marew Alemenhew, Gashaw Andargie Biks, Ansha Nega

**Affiliations:** 1 Department of Human Nutrition, Institute of Public Health, College of Medicine and Health Sciences, University of Gondar, Gondar, Ethiopia; 2 Department of Nursing, College of Medicine and Health Sciences, University of Gondar, Gondar, Ethiopia; 3 Department of Nursing, Teda Health Science College, Gondar, Ethiopia; 4 Department of Health Service Management and Heath Economics, Institute of Public Health, College of Medicine and Health Sciences, University of Gondar, Gondar, Ethiopia; 5 Department of Environmental and Occupational Health and Safety, Institute of Public Health, College of Medicine and Health Sciences, University of Gondar, Gondar, Ethiopia

**Keywords:** Food hygiene practice, Food handlers, Restaurants, Ethiopia

## Abstract

**Background:**

Morbidity and mortality rates of food borne diseases are consistently highest in African due to poor food handling and sanitation practices. Thus, the study aimed to assess food handling practice and associated factors among food handlers of Restaurants in Bahir Dar Town, northwest, Ethiopia. A cross-sectional study was conducted from December, 7/2012 to January, 2/2013 among food handlers working in 106 restaurants in Bahir Dar Town. A structured questionnaire composed of socio-demographic factors, food safety knowledge, working environmental characteristics and food hygiene practice of food handlers was employed to collect the data via interviewing and observations. Binary logistic regression model was fitted to assess factors associated with food hygiene practice after multi-collinearity and outlier were checked and data was clean. Both crude odds ratio (COR) and adjusted odds ratio (AOR) were estimated to show the strength of association. In multivariate analysis, variables with a *P* value of ≤ 0.05 were considered as statistical significant.

**Results:**

About 67.6% [95% confidence interval (CI) 58.8, 76.4] of food handlers had good food hygiene practice, whereas 32.4% of food handlers had poor food hygiene practice. The odds of having good food hygiene practice was higher among food handlers who had received food safety training [AOR: 4.7, 95% CI 1.7, 12.8], had formal education [AOR: 6.4, 95% CI 3.5, 11.5] and work experiences greater than 2 years [AOR: 3.4, 95% CI 1.8, 6.4]. At last, food handlers working in restaurants which had piped fountains for hand wash were 2.1 times more likely to have good food hygiene practice[AOR: 2.1, 95% CI 1.1, 3.8].

**Conclusion:**

In this study, the overall food hygiene practice of food handlers is not to the acceptable level. Therefore, endeavors ought to be reinforced to improve food hygiene practices of food handlers through intervention programs such as training and education. Also emphasis should be given on the accessibility of piped fountains for the better food handling practice of food handlers.

## Background

Globally, every year billions of people are at high risk and millions fall ill; many die as a result of consuming unsafe food [1]. More than 200 known diseases have been transmitted through unsafe food [2]. As a result, around 2.0 billion illnesses are associated with food borne diseases [1]. Concerning low income countries, food born disease causes 2 million deaths. Also, it is related to high rate of hospitalizations and treatment cost [1, 3, 4]. In particular, the highest rate of morbidity and mortality rates are consistently reported in African. Poor food handling and sanitation practices, inadequate food safety laws, weak regulatory systems, lack of financial resources to invest on safety equipments and poor literacy status of food-handlers are some of the attributors augmenting the adverse consequences of food born disease [1, 3–5].

Obviously, food handlers play a critical role in ensuring food hygiene in the food establishments [6, 7]. Nevertheless, 10–20% of food-borne diseases are due to contamination of food by food handlers [8]. This is mainly due to poor hygienic status of food handlers, improper cooking procedures and unsafe storage and handling of food and equipments, which paves the way for pathogens to come into contact with food and cause illness in consumers [9–12]. Cognizant of these problems, the World Health Organization (WHO) established five keys to safer food manual to educate food handlers about their responsibilities for food safety [10]. Therefore, understanding of food safety procedures and potential factors that cause food borne illness are critical for all food handlers in the prevention and control of food borne illness [10, 13]. Different factors associated with the level of food hygiene practice among food handlers have been identified in different study settings. Accordingly, advanced age [14], divorced marital status [15], good knowledge on food safety and formal education [16, 17], work experience [14], food safety training [18] and monthly income [15] are positively associated with food hygiene practice. Furthermore, the environmental factors, such as toilet facility, liquid and solid waste management, water supply, and infestation of vectors are found related to the level of food hygienic practice of food handlers [15, 19].

In Ethiopia there is no reliable information showing magnitudes of food borne illness related to inappropriate food handling s in food establishments. However, there are noticeably high insanitary conditions in food establishments, including in the capital city, Addis Ababa.

On the other hand, helminthes, dysentery and diarrheal disease are among the top 10 leading causes of outpatient health service visits, which can be effectively prevented with provision of safe food and adequate water supply [20–23]. Bahir Dar Town, the capital of Amhara regional state is showing rapid increase in urbanization, industrialization, one of the tourist destinations, and center of training and conference with the resultant increment in the number of restaurants. Accordingly, the number of people eating outside their homes is expected to increase, and this can increase the risk of food borne diseases. Therefore, providing evidence on food handling practice and its determinant will have a crucial role in improving food hygienic practices for food handlers working in restaurants. However, literatures are limited in Ethiopia, especially in the study area. Therefore, this study aimed to assess food handling practice and associated factors among food handlers of restaurants in Bahir Dar Town, northwest Ethiopia.

## Methods

### Study design and setting

A cross-sectional study was conducted from December 2012 to January 2013 among food handlers working in 106 restaurants in Bahir Dar Town. Bahir Dar Town, the capital of Amhara National Regional State, is located 565 kms from Addis Ababa, the capital city of Ethiopia. According to 2010 census, the population size of the town was estimated at 256,999 [24].

### Study participants and sampling procedure

All food handlers, in the kitchen or serving in dinning, working in 106 restaurants of Bahir Dar Town were included in the study. Sample size was determined using a single population proportion formula by considering the following assumptions; expected prevalence of good food hygiene practice as 50, 95% level of confidence and 5% margins of error(w). Adding 10% non-response the final minimum sample size was 422. Regarding to the sampling technique, before hand, the total list of restaurants (110) and food handlers (782) were obtained from trade and industry Office of Bahir Dar Town. Then, the number and list of food handlers in each restaurant was obtained from each restaurant owner or manager during data collection. Food handlers were selected based on the proportion (422/782 * 100 = 53%) to the size of food handlers in each restaurant. A minimum of one food handlers from kitchen or serving in the dining, per restaurant, was selected. But, in the presence of more than one food handlers in a single food preparation and/or service area, selection was done by lottery method. At the end of data collection the total number of restaurants and food handlers in the Town was 106 and 789, respectively. Later on, 417 food handlers working in restaurants were included in the analysis.

### Data collection tools and procedure

A structured questionnaire composed of socio-demographic factors, food safety knowledge, working environment characteristics and food hygiene practice was employed to collect the data via face to face interview and observation. The questionnaire was designed from standardized food and drink establishments’ inspection checklist in Ethiopia and by reviewing different literatures. To maintain its consistency, the questionnaire was originally prepared in English, and translated to Amharic, then retranslated to English. Seven diplomas in public and environmental health and two BSc environmental health experts were recruited as data collector and field supervisor, respectively. One day training regarding the objective of the study, interview and inspection techniques, and confidentiality of information was given to data collectors and supervisors. Shortly after, the questionnaire was pre-tested on 5% of the total sample out of the study area. To maintain the quality of data, the investigators and supervisors were carried out regular supervision, spot-checking, and reviewing the completed questionnaire on daily basis.

### Operational definitions and study variables

The level of food hygiene practice was determined by using 17 food hygiene practice questions complemented with direct observation. The food hygiene practice was computed with a maximum score of seventeen. By considering the mean score (12), the food hygiene practice of food handlers was categorized as poor if their score was below twelve, otherwise good practice if their score was greater or equal to twelve. The independent variables included in the study were socio-demographic characteristics (age, sex, religion, marital status, educational status, work experience, work responsibility, working hours and training), knowledge on food safety and work environment related characteristics (toilet facility, source of water, type of hand washing facility and building of ownership). Regarding to food safety knowledge, ten questions were used to determine the food handler’s knowledge about food safety. Finally, by considering the mean score as 6, the food handlers’ knowledge was categorized as poor if their score was less than six, otherwise good knowledge if they score greater than or equal to six.

### Data analysis

Data were checked for completeness, edited, coded and entered into the EPI-info version 3.5.3 statistical software, and exported to SPSS version 16 for analysis. Descriptive statistics were carried out for variables in the study using frequency tables, percentage, standard deviation and mean. Initially, cleaning and checking for multi-collinear and outlier variable was done. Then, binary logistic regression model was fitted to assess factors associated with food hygiene practice. In bivariate analysis (crude odds ratio) variables with a P value of ≤ 0.2 were entered into multivariate analysis to control the possible effect of confounders. Both crude odds ratio (COR) and adjusted odds ratio (AOR) were estimated to show the strength of association. Besides, Hosmer and Lemeshow goodness of fit test was checked and it was 0.97 indicating the model well fits the data. In multivariate analysis, variables with a P value of ≤ 0.05 were declared as statistically significant.

## Result

### Socio-demographic characteristics

A total of 417 food handlers were included in the study with the response rate of 98.8%. Above three-fourths (77.7%) of food handlers were females. More than one-third (39.3%) of the food handlers had primary school education. However, majority (83%) of participants did not take a food safety training (Table 1).

**Table 1:** Socio-demographic characteristics of food handlers working in the restaurants of Bahir Dar Town, northwest, Ethiopia (n = 417)

Characteristics	Frequency	Percent
Sex		
Male	93	22.3
Female	324	77.7
Age		
16–20	143	34.3
21–30	255	61.2
> 30	19	4.5
Education status		
No read and write	51	12.2
Read and write	31	7.5
Primary school	164	39.3
Secondary school	133	32.9
College/university	38	9.1
Marital status		
Married	53	12.7
Single	352	84.4
Divorced	12	2.9
Religion		
Orthodox	376	90.1
Muslim	37	8.9
Protestant	4	1
Work responsibility		
Cooker	174	41.7
Waiter	243	58.3
Work experience (years)		
< 2	290	69.5
≥ 2	127	30.5
Work hours (h)		
≤ 8	152	36.5
> 8	265	63.5
Food safety training		
Yes	71	17
No	346	83
Certification (n = 71)		
Yes	26	36.6
No	45	63.4

### Food safety knowledge of food handler

Out of 417 food handlers, majority 342 (82%) had good food safety knowledge (mean score ≥ 6). Vast majority 373 (89.4%) of food handlers had heard about food borne diseases. Mass media was the most common 252 (67.6%) source of information, followed by sanitarian during inspection 200 (53.6%). The proportion of food handlers who believed that food borne diseases are caused by germs was 357 (95.7%). However, the study demonstrated that substantial proportion (81.8%) of food handlers in Bahir Dar Town restaurants were not aware of the correct temperature for a refrigerator to keep the food safer (Table 2).

**Table 2:** Food safety knowledge of food handlers in Bahir Dar Town, northwest, Ethiopia

Knowledge questions	Frequency	Percent
Have you ever heard about food borne disease?		
Yes	373	89.4
No	44	10.6
Who is your source of information about food borne disease?		
Health center	126	33.8
Sanitarian inspection	200	53.6
Mass media	252	67.6
School	115	30.8
Customers, friends and family	42	11.3
What is the cause of food borne disease?		
Germs	357	95.7
Chemicals	205	55
Food borne disease is transmitted by		
Contaminated food	340	91.2
Contaminated water	217	58.2
Vectors	237	63.5
What is the reason for food contamination?		
Dirty hands	318	85.3
Dirty working environment/area	352	94.4
Using of contaminated water	299	80.2
Unclean/dirty utensils	292	78.3
Infected food handlers	211	56.6
The correct temperature for refrigerator is 1–5 °C		
Yes	76	18.2
No	341	81.8
The temperature danger zone for potentially hazardous food is 5–60 °C		
Yes	48	11.5
No	369	88.5
Does raw meat transmit disease?		
Yes	355	85.1
No	62	14.9
Does raw milk transmit disease?		
Yes	404	96.9
No	13	3.1
Do raw vegetables transmit disease?		
Yes	316	75.8
No	101	24.2
Personal hygiene of food handler can prevent food borne disease		
Yes	396	95
No	21	5
Knowledge of food safety		
Good	342	82
Poor	75	18

### Working environment and food hygiene practice of food handlers

#### Characteristics of food handlers

All food handlers (99.8 and 100%, respectively) were working in restaurants where private piped water and toilet facility are available (Table 3).

**Table 3:** Working environmental characteristics of food handlers, in Bahir Dar Town, northwest Ethiopia

Variables	Frequency	Percent
Ownership of building		
Owned	224	53.7
Rented	193	46.3
Source of water		
Private pipe	411	98.6
Others^a^	6	1.4
Waste disposal		
Septic tank	278	66.7
Open space	21	5
Water drainage	97	23.2
Availability of latrine		
Yes	417	100
No	0	0
Availability of kitchen		
Yes	416	99.8
No	1	0.2
Type of hand washing facility		
Piped fountains	296	71
Welded metals	48	11.5
Jug or discarded object	73	17.5

^a^Pipe shared, pipe from neighbor

About 67.6% [95% CI 58.8, 76.4] of food handlers had good food hygiene practice. Two-third (65.7 and 64.3%, respectively) of food handlers wearied outer garments cleaned and sanitized work surfaces after each task had good food hygiene practice. However, below one-third (29.5%) of food handlers who covered their hair while working had good food hygiene practice (Table 4).

**Table 4:** Food hygiene practice among food handlers in restaurants of Bahir Dar Town, northwest, Ethiopia

Food hygienic practices	Good (%)	Poor (%)
Does the food handler wear outer garments/gown during visit?		
Yes	274 (65.7)	63 (15.1)
No	8 (1.9)	72 (17.3)
Cleanness of outer garments?		
Yes	203 (48.7)	51 (12.2)
No	62 (14.9)	101 (24.2)
Does the food handlers hair covered while working in food service establishments during visit?		
Yes	123 (29.5)	39 (9.4)
No	259 (62.1)	96 (23.0)
Does the food handler’s finger nail short trimmed and clean?		
Yes	247 (59.2)	85 (20.4)
No	35 (8.4)	50 (12)
Does food handler wear any jewelry or ring on hand at time of visit?		
Yes	263 (63.1)	76 (18.2)
No	19 (4.6)	59 (14.1)
Does food handler clean and sanitize work surfaces after each task today?		
Yes	268 (64.3)	52 (12.5)
No	14 (3.4)	83 (19.9)
Does food handler use soap/detergent for washing dishes?		
Yes	271 (65)	104 (25)
No	11 (2.6)	31 (7.4)
Does the food handler use hot water for washing dishes?		
Yes	117 (28)	15 (3.6)
No	165 (39.6)	120 (28.8)
Does the food handler wash cutting surfaces/knife/with soap/belch/after using it for cutting raw meat or chicken?		
Yes	196 (47.1)	11 (2.6)
No	86 (20.6)	124 (29.7)
Does the food handler wash his/her hands with soap and water before working with food?		
Yes	249 (59.7)	65 (15.6)
No	33 (7.9)	70 (16.8)
Does the food handler wash his/her hands with soap and water after visiting a latrine?		
Yes	268 (64.3)	99 (23.8)
No	14 (3.4)	36 (8.6)
Does the food handler drink or eat food while serving or preparing food?		
Yes	280 (67.2)	88 (21)
No	2 (0.5)	47 (11.3)
Does the food handler kept ready-to-eat foods in a clean container and covered properly?		
Yes	257 (61.6)	40 (9.6)
No	25 (6)	95 (22.8)
Does the food handler stored food utensils in well-arranged manner in shelf or cupboard?		
Yes	243 (58.3)	21 (5)
No	39 (9.4)	114 (27.4)
Does the food utensils free of dust particles, finger paint and other marks?		
Yes	278 (66.6)	58 (13.8)
No	4 (1)	77 (18.5)
Does the food handler use a separate clean utensil for each food item?		
Yes	277 (66.4)	52 (12.5)
No	5 (1.2)	83 (19.9)
Does the food handler store raw food item in an area separate from cooked food?		
Yes	268 (64.2)	31 (7.4)
No	14 (3.4)	104 (25)

#### Factors associated with food hygiene practice

In the bivariable analysis, age, education, food safety training, work responsibility, work experience and type of hand washing were associated with P value of ≤ 0.2. On the other hand, the multivariate analysis revealed that food safety training, education, work experience, and type of hand washing were significantly and independently associated with food hygiene practice of food handlers.

With this regard, the odds of having good food hygienic practice were 4.7 times [AOR: 4.7, 95% CI 1.7, 12.8] higher among food handlers who received food safety training. Likewise, the odds of having good food hygiene practice were higher among food handlers who had formal education [AOR: 6.4, 95% CI 3.5, 11.5]. The likelihood of having good food hygienic practice was 3.4 folds [AOR: 3.4, 95% CI 1.8, 6.4] higher among food handlers who had work experiences of greater than or equal to 2 years. Finally, the type of hand wash, piped fountains, was associated with good food hygiene practice [AOR: 2.1, 95% CI 1.1, 3.8] (Table 5).

**Table 5:** Factors associated with food hygienic practice among food handlers working in restaurants of Bahir Dar Town, northwest, Ethiopia

Variables	Food hygienic practice		Crude odds ratio (95% CI)	Adjusted odds ratio (95% CI)
	Good	poor		
Educational status				
No formal education	24	58	1	1
Formal education	258	77	8.1 (4.72, 13.88)	6.4 (3.5, 11.5)
Work responsibility				
Cooker	108	66	1	1
Waiter	174	69	1.5 (1.1, 2.3)	1.3 (0.8, 2.2)
Work experience (years)				
< 2	172	118	1	1
≥ 2	110	17	4.4 (2.5, 7.8)	3.4 (1.8, 6.4)
Training				
Yes	66	5	7.9 (3.1, 20.2)	4.7 (1.7, 12.8)
No	216	130	1	1
Type of hand washing				
Piped fountains	208	88	2.3 (1.4, 3.9)	2.1 (1.1, 3.8)
Metal welded	11	37	0.3 (0.3, 1.4)	0.6 (0.3, 1.4)
Jug or discarded object	37	36	1	1

## Discussion

This study investigated the status of food hygiene practice and associated factors among food handlers. The reported illustrated that 67.6% of the food handlers had good food hygiene practice. This finding was consistent with a study conducted in Mekelle University student’s cafeteria, Ethiopia (63.9%) [12]. On the other hand, the good food hygiene practice in this study was higher compared with study conducted in Dangila Town, Ethiopia (52.5%) [15]. This discrepancy might be due to better inspection and regulation systems in the current study because of Bahir Dar Town is the capital of Amhara region, center of tourist destination, training and conference center compared to the later report. Moreover, this result was higher than a study conducted in Nigeria (56.3%) [25]. The observed difference might be due to sample size, operational definition, demographic and regulatory systems variations.

The odds of having good food hygienic practice were 4.7 times higher among food handlers who received food safety training. This finding was supported by the earlier studies [18, 26, 27]. Training enhances food handlers’ awareness on food borne diseases [10, 26]. In addition, food hygiene training could enable food handlers to better understand and fulfill their responsibilities and exercise skills [26].

This study also showed that the odds of good food hygiene practice were higher among food handlers who had formal education compared to no formal education. The study conducted in Ethiopia [16, 28], Malaysia [27] and Nigeria [25] revealed the importance of education for food handlers to ensure food safety. Obviously, education helps to augment knowledge thereby to develop skill of food handlers to work according to standard procedures to maintain food hygienic/safety [13].

As well, the result of this study showed that the practice of food hygiene was 3.4-fold more satisfactory in food handler’s having work experience greater or equal 2 years than food handlers having working experience less than 2 years. A study conducted in Iowa State University Hotel and Restaurant, practice scores increased as food handler’s works Services advances [14]. This might be due o the fact that behaviors can be learned through repeated practice, therefore experienced food handlers are in better position to acquired skills on food hygiene.

Finally, food handlers working in restaurants with piped fountains for hand wash were 2.05 times more likely to have good food hygiene practice compared to those working in establishments which had a jug or discarded object. The possible reason might be those food handlers working in restaurants the type of hand washing were piped fountains may better to keep their personal hygiene, clean the working environment easily which in turn enables to attain good food hygienic practice.

## Conclusions

In this study, the overall food hygiene practice of food handlers is not to the acceptable level. Therefore, endeavors ought to be reinforced to improve food hygiene practices of food handlers through intervention programs such as training and education. Also emphasis should be given ensure availability of piped fountains in each restaurant.

## Abbreviations

WHO: World Health Organization
AOR: adjusted odds ratio
COR: crude odds ratio
CI: confidence interval
SD: standard deviation
